# Supplementary material for: Phased Assembly of Neo-Sex Chromosomes Reveals Extensive Y Degeneration and Rapid Genome Evolution in Rumex hastatulus
Source: Mol Biol Evol. 2024 Apr 12;41(4):msae074. doi: 10.1093/molbev/msae074 (PMC11057207; doi:10.1093/molbev/msae074)
Supplement: msae074_Supplementary_Data [file msae074_supplementary_data.pdf]

Supplementary Material

Phased assembly of neo-sex chromosomes reveals extensive Y degeneration and rapid genome evolution in *Rumex hastatulus*

Bianca Sacchi[1*], Zoë Humphries[1*], Jana Kružlicová[2], Markéta Bodláková[2], Cassandre Pyne[1], Baharul Choudhury[1,3], Yunchen Gong[4], Václav Bačovský[2], Roman Hobza[2], Spencer C.H. Barrett[1], and Stephen I. Wright[1,4]

Table S1- Genome Assembly Statistics

Assembly	Total Assembly Length	Contig N50	Contig L50	Contig number	Scaffold N50	Scaffold L50	Scaffold N90	Scaffold L90
R. salicifolius	586,431,610	53,106,915	5	19	56,809,946	5	51,010,208	9
R. hastatulus haplotype A	1,510,206,782	6,283,184	73	361	466,657,150	2	168,693,024	4
R. hastatulus haplotype B	1,719,830,090	5,620,551	92	468	343,398,141	3	166,046,2686	5

Table S2 - BUSCO results

**Genome**	**OrthoDB**	**Complete, single copy**	**Complete, duplicated**	**Fragmented**	**Missing**
R. salicifolius assembly	Eukaryota	227 (89%)	27 (10.6%)	0	1 (0.4%)
R. hastatulus haplotype A assembly	Eukaryota	223 (87.5%)	30 (11.8%)	0	2 (0.7%)
R. hastatulus haplotype B assembly	Eukaryota	224 (87.8%)	30 (11.8%)	0	1 (0.4%)
R. salicifolius assembly	Embryophyta	1528 (94.7%)	39 (2.4%)	20 (1.2%)	27 (1.7%)
R. hastatulus haplotype A assembly	Embryophyta	1476 (91.4%)	77 (4.8%)	20 (1.2%)	41 (2.6%)
R. hastatulus haplotype B assembly	Embryophyta	1463 (90.6%)	71 (4.4%)	26 (1.6%)	54 (3.4%)

R. salicifolius annotation	Eukaryota	211 (82.7%)	27 (10.6%)	10 (3.9%)	7 (2.8%)
R. hastatulus haplotype A annotation	Eukaryota	200 (78.4%)	23 (9.0%)	16 (6.3%)	16 (6.3%)
R. hastatulus haplotype B annotation	Eukaryota	211 (82.7%)	25 (9.8%)	6 (2.4%)	13 (5.1%)
R. salicifolius annotation	Embryophyta	1379 (85.4%)	44 (2.7%)	47 (2.9%)	144 (9.0%)
R. hastatulus haplotype A annotation	Embryophyta	1312 (81.3%)	57 (3.5%)	81 (5.0%)	164 (10.2%)
R. hastatulus haplotype B annotation	Embryophyta	1331 (82.5%)	50 (3.1%)	58 (3.6%)	175 (10.8%)

Table S3 – Non-syntenic orthologs

Region	Number of non-syntenic orthologs unique to haplotype A [1]	Number of non-syntenic orthologs unique to haplotype B [2]
A1	14	22
A2	11	34
A4	6	6
New sex-linked region	1	6
Old sex-linked region	13	155
Pseudo-autosomal regions	2	15

1. Non-syntenic orthologs unique to haplotype A (X-bearing) are syntenic between haplotype B (Y-bearing) and R. salicifolius only.
2. Non-syntenic orthologs unique to haplotype B are syntenic between R. salicifolius and haplotype A only.

Table S4 - TE count chi-square

Region	Linkage	*p-value*	Expected	X observed	Y observed	Y/X ratio
entire	New	1.332333e-71	88431.5	92194	84669	0.9183786
entire	Old	0.00000e+00	299641.5	158443	440840	2.7823255
entire	PAR	2.220696e-11	160351.5	158457	162246	1.0239118
upstream	New	8.139606e-02	951.0	913	989	1.0832421

upstream	Old	5.521570e-27	1608.0	1303	1913	1.4681504
upstream	PAR	1.598126e-05	5035.5	4819	5252	1.0898527
downstream	New	5.394717e-06	1015.5	913	1118	1.2245345
downstream	Old	2.074799e-41	1774.5	1373	2176	1.5848507
downstream	PAR	2.162392e-16	5045.5	4633	5458	1.1780704
exon	New	3.411261e-01	565.0	549	581	1.0582878
exon	Old	5.152438e-19	1154.0	940	1368	1.4553191
exon	PAR	2.187631e-20	1703.0	1973	1433	0.7263051
intron	New	2.398108e-02	812.5	767	858	1.1186441
intron	Old	1.104104e-19	1334.5	1100	1569	1.4263636
intron	PAR	6.717053e-01	4454.0	4474	4434	0.9910595

Table S5 – XYY male haplotype B gene loss results

Region	Number of genes with syntenic orthologs in outgroup [1]	Proportion of genes completely absent in haplotype B [2]	Proportion of genes partially absent in haplotype B [3]
A1	2786	0.0004	0.0262
A2	2833	0.0039	0.0289
A4	1535	0.0033	0.0319
New X	252	0.0000	0.0278
Old X	745	0.2107	0.1275
PAR1	793	0.0076	0.0303
PAR2	1295	0.0015	0.0409

1. Total number of genes in haplotype A sharing a syntenic ortholog with the outgroup *R.* salicifolius, with or without orthologs in haplotype B.
2. Proportion of haplotype A genes that are not orthologous haplotype B genes and do not return any BLAST hits when aligned to the haplotype B genome assembly.
3. Proportion of genes with partial BLAST hits.

Table S6 – XYY male haplotype A gene loss results

Region	Number of genes with syntenic orthologs in outgroup [1]	Proportion of genes absent in maternal haplotype [2]
A1	2702	0.0248
A2	2714	0.0247
A4	1451	0.0207
NewY1	145	0.0069
NewY2	96	0.0208
OldY1	206	0.0146
OldY2	280	0.0250

PAR1	755	0.0265
PAR2	1236	0.0218

1. Total number of genes in haplotype B sharing a syntenic ortholog with the outgroup *R. salicifolius*
2. Proportion of genes in 1 that do not share orthology with haplotype A

Table S7. The list of used primers of candidate satellites

Primer	Sequence (5´-3´)	Product size	Annealing temperature (°C)
**Cl12F**	AGTTCGTTGTCCAATATACTCGTT	117	58
**Cl12R**	TGTGAAAACGTACCGAACATGT		
**Cl133 F**	GTATAAAAACCGTAACCGTG	66	53
**Cl133 R**	GCTCGAAATAGTGTAATCAAAC		
**Cl134 5S F**	CCACTGATATATTGACCGCTCGA	265	60
**Cl134 5S R**	AAAGGCAATATCTCATCCGATAGT		
**Cl135 F**	CAATTTCATGACGGTTAATAACG	1136	55
**Cl135 R**	CACGGTTACGGTTTTTATAC		
**Cl162F**	GCTCAAAATTTTGTTGCTCTCC	80	60
**Cl162R**	TGGTGACTTAAAACATCAGAGCAAC		
**Cl168 F**	TGTACAATGGTAGAAATCGGGTC	170	55
**Cl168 R**	CAAATTTTGTTCAATCTC		
**Cl86 F**	CACTTGCCCGATGGAAACGGC	170	55
**Cl86 R**	CCTTGTTCATCCGTTTCCGGTG		

Table S8. The composition of the hybridization mixture used in FISH

Hybridization mixture Stringency 87%	Volume per one slide (µl)
100% formamide	10
50% dextran sulphate	4
20xSSC	0.5
ddH2O	X (to final volume)
DNA probe	1 (for each cluster)
Final volume 20 ml	

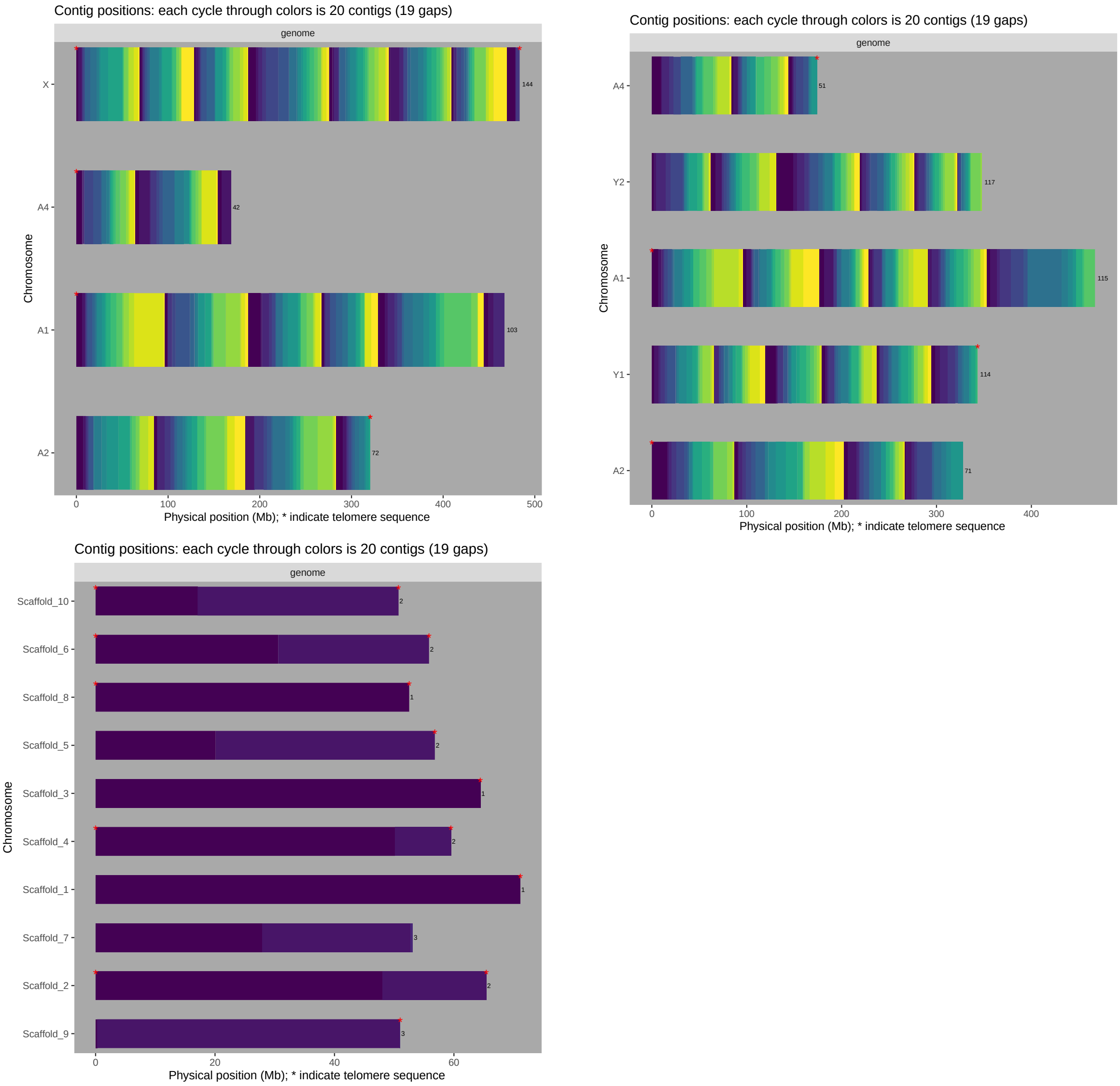

Figure S1. Contig size in *Rumex hastatulus* XYY, Haplotype A, haplotype B, and *R. salicifolius* genome assemblies. Stars correspond to locations of telomere array sequence.

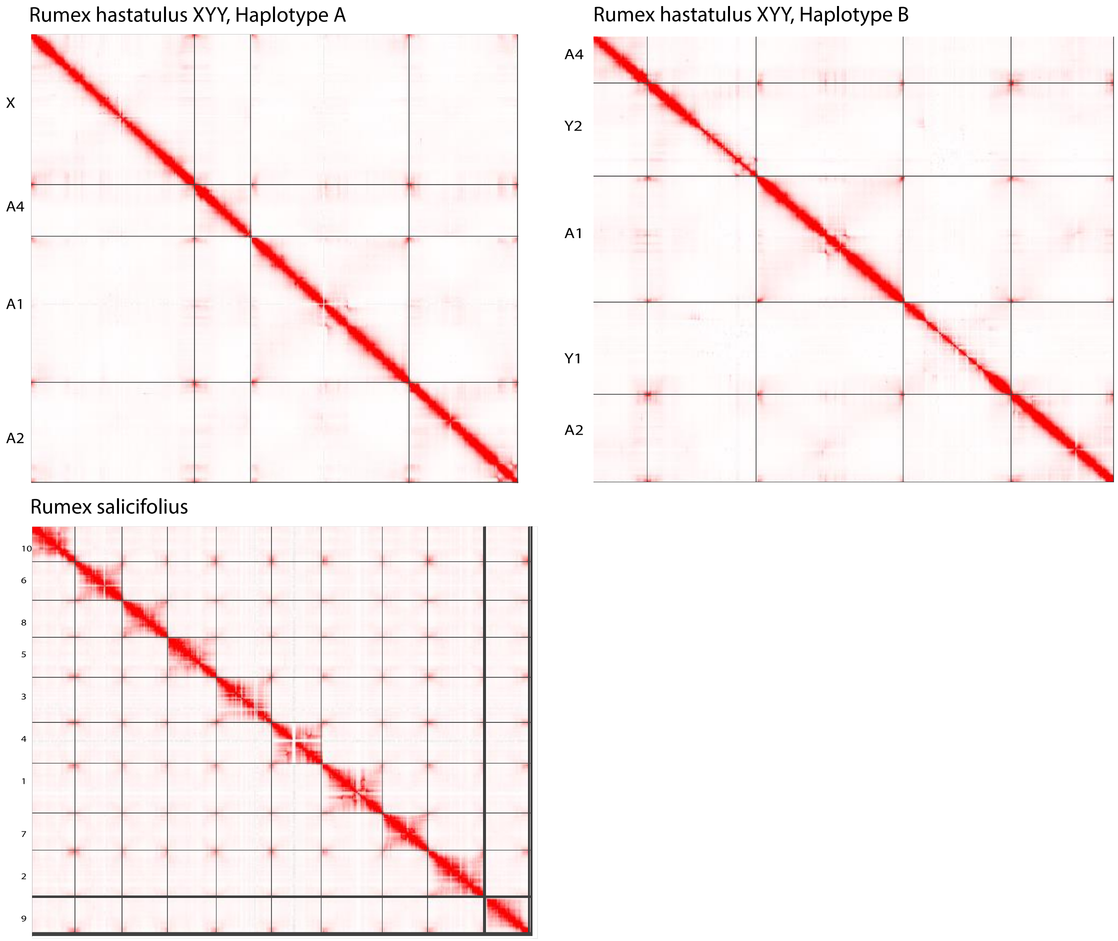

Figure S2.

Omni-C contact maps for phased assemblies of *Rumex hastatulus* and the assembly of *R. salicifolius*

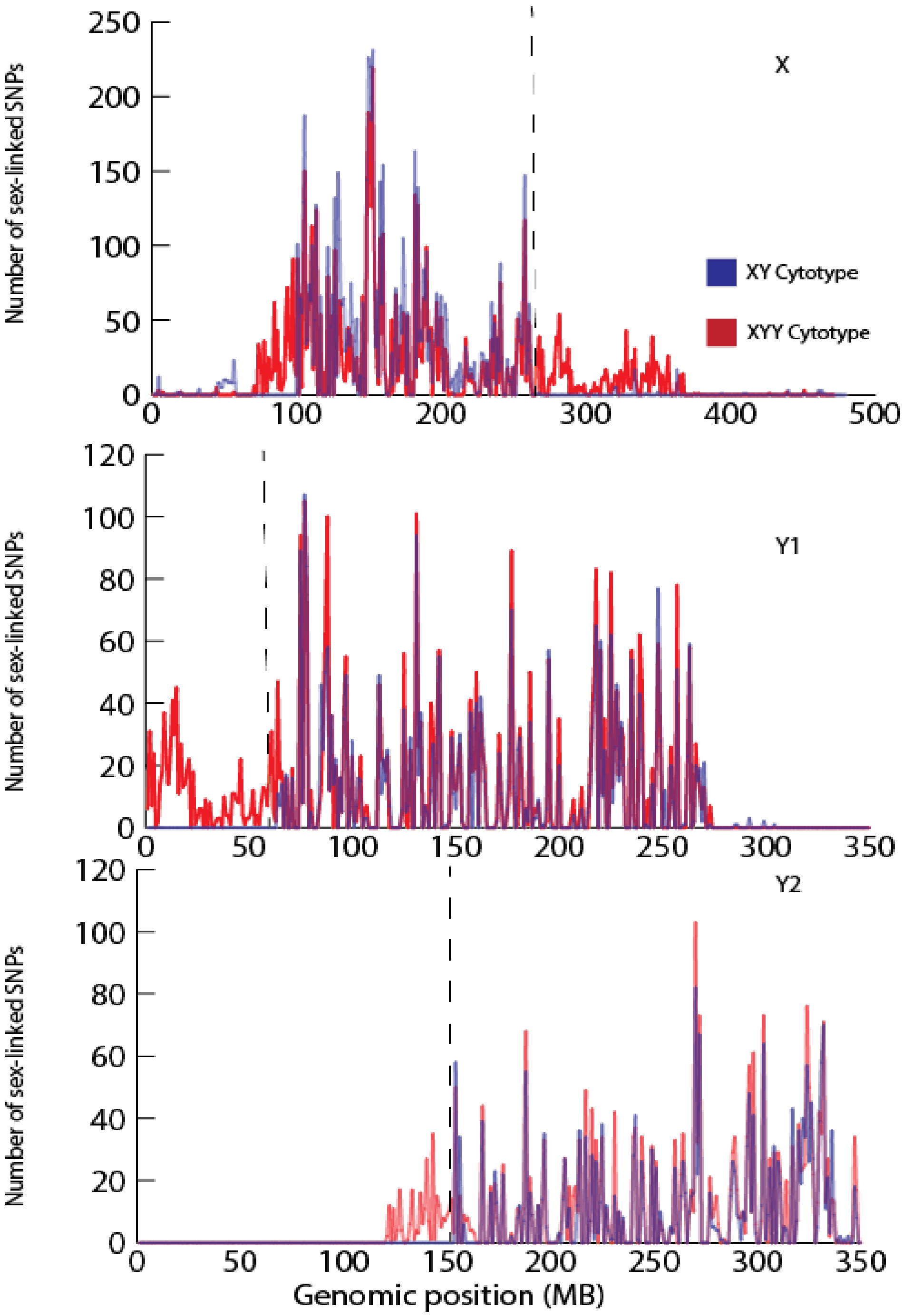

Figure S3. Sex-linked SNP patterns along the XYY cytotype's X, Y1 and Y2 chromosomes. Density of sex-linked SNPs in 1 MB windows in the XY and XYY cytotypes. Dashed line indicates the inferred breakpoint between the old X (Y) chromosome and the neo-X (neo-Y) region.

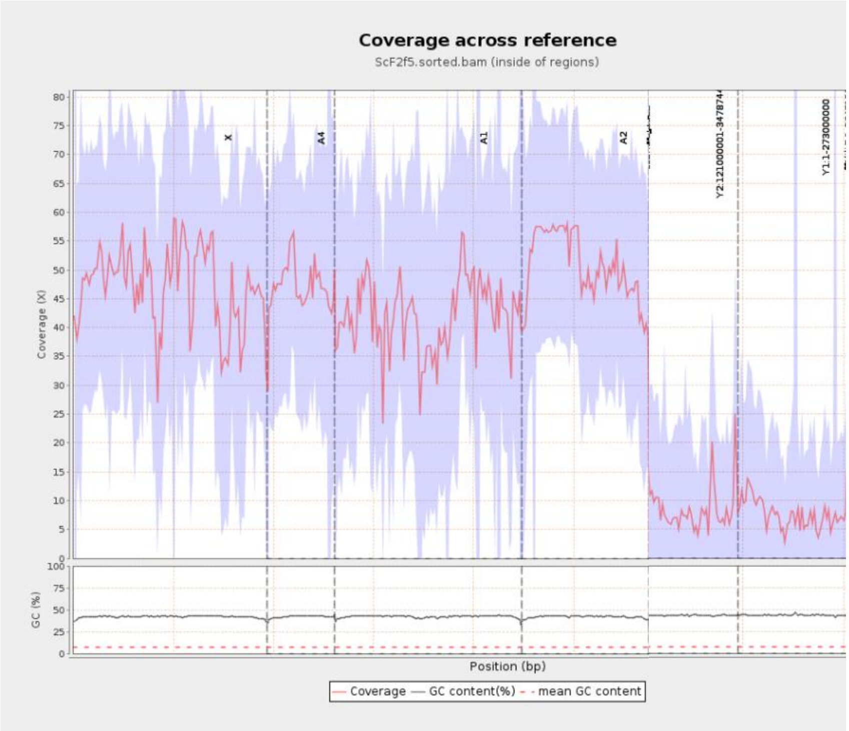

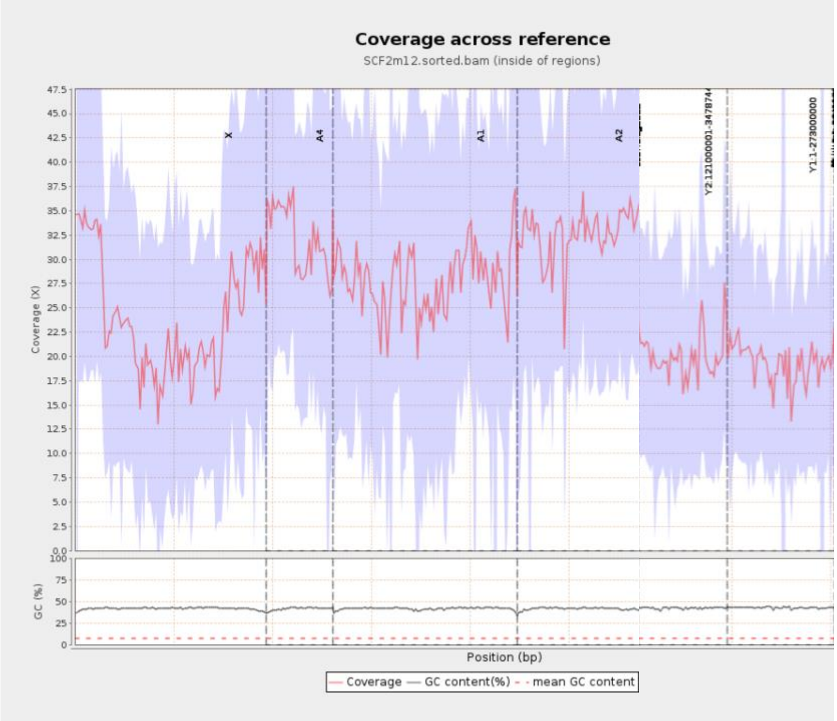

Figure S4. Depth of genomic reads from a female (top panel) male (bottom panel) XYY individual aligned to reference containing X, Y1, Y2 and autosomes. The high coverage regions on either end of the X in the male coverage plot corresponds to the pseudoautosomal regions. Mean and standard deviation of coverage is plotted in 400 windows across the genome. Small unassembled scaffolds have been removed from the plot.

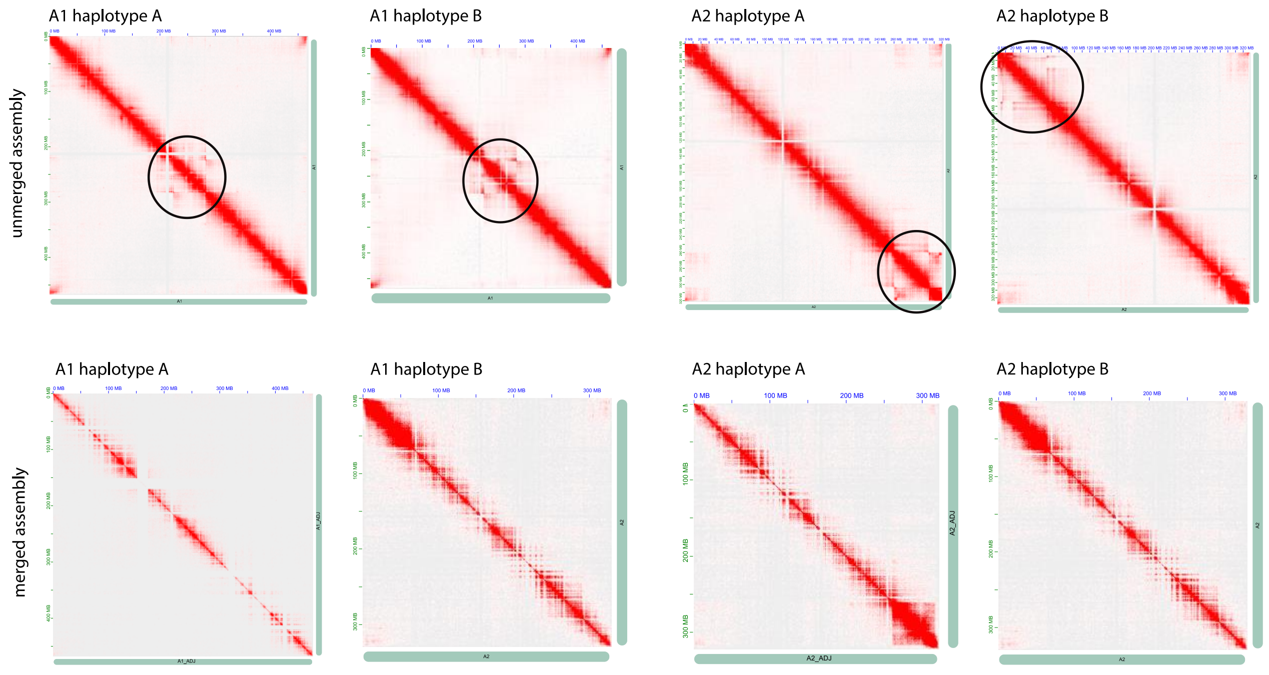

Figure S5.
OmniC contact maps for putative inverted regions. Top row shows OmniC maps for individual haplotype assemblies. The inverted regions are circled. Bottom row shows contact maps for combined reference including both haplotypes.

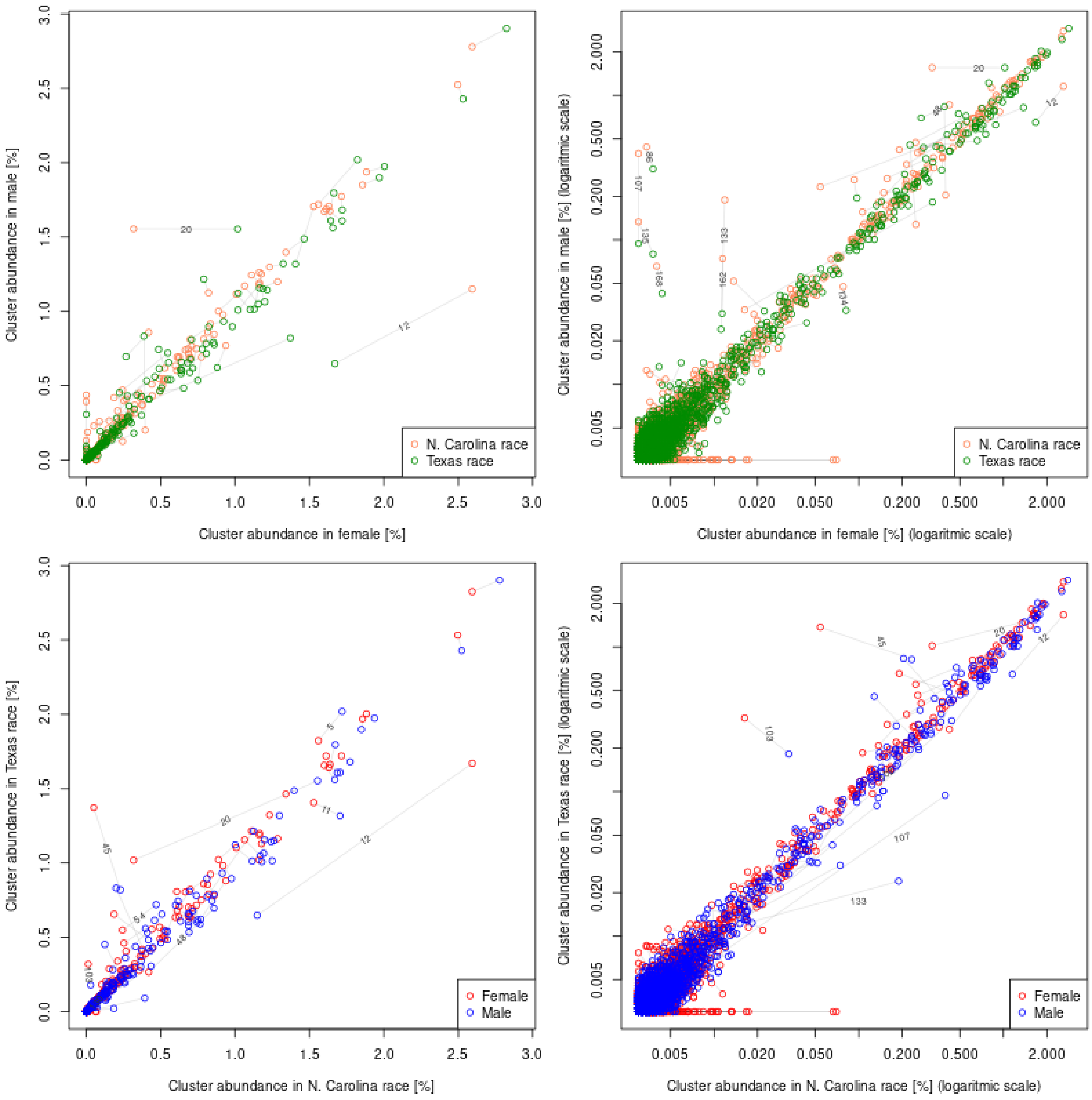

Figure S6. Repeat cluster abundance in XYY and XY cytotype. Comparison of males and female (top) and XYY/XY cytotypes (bottom). Right plots are identical to left plots in logarithmic scale.

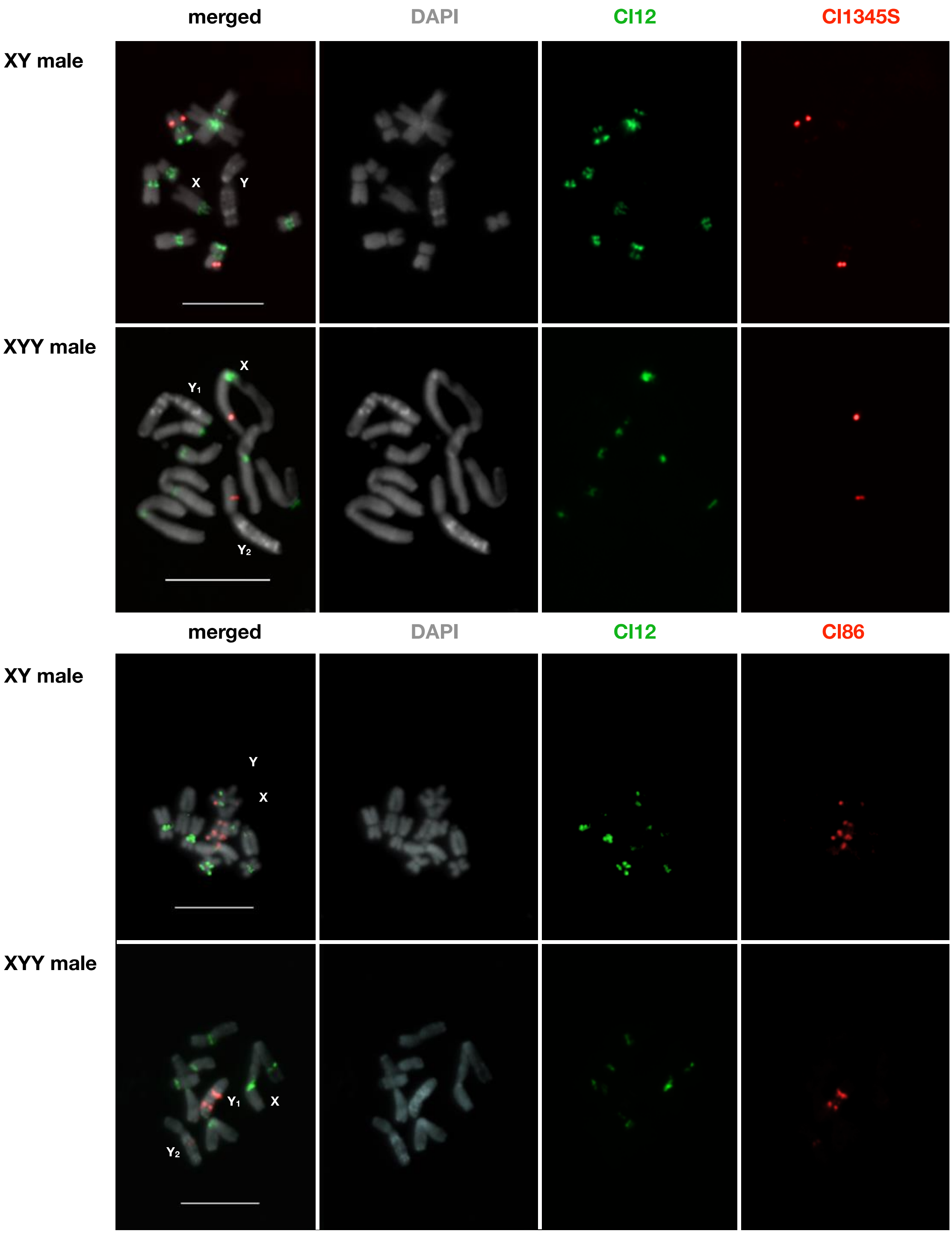

Figure S7. The localization of Cl12 (green), Cl134 5S (red-top) and Cl86 (red bottom) in XY and XYY males cytotypes. Metaphase chromosomes counterstained with DAPI (grey). Scale bar = 10 mm

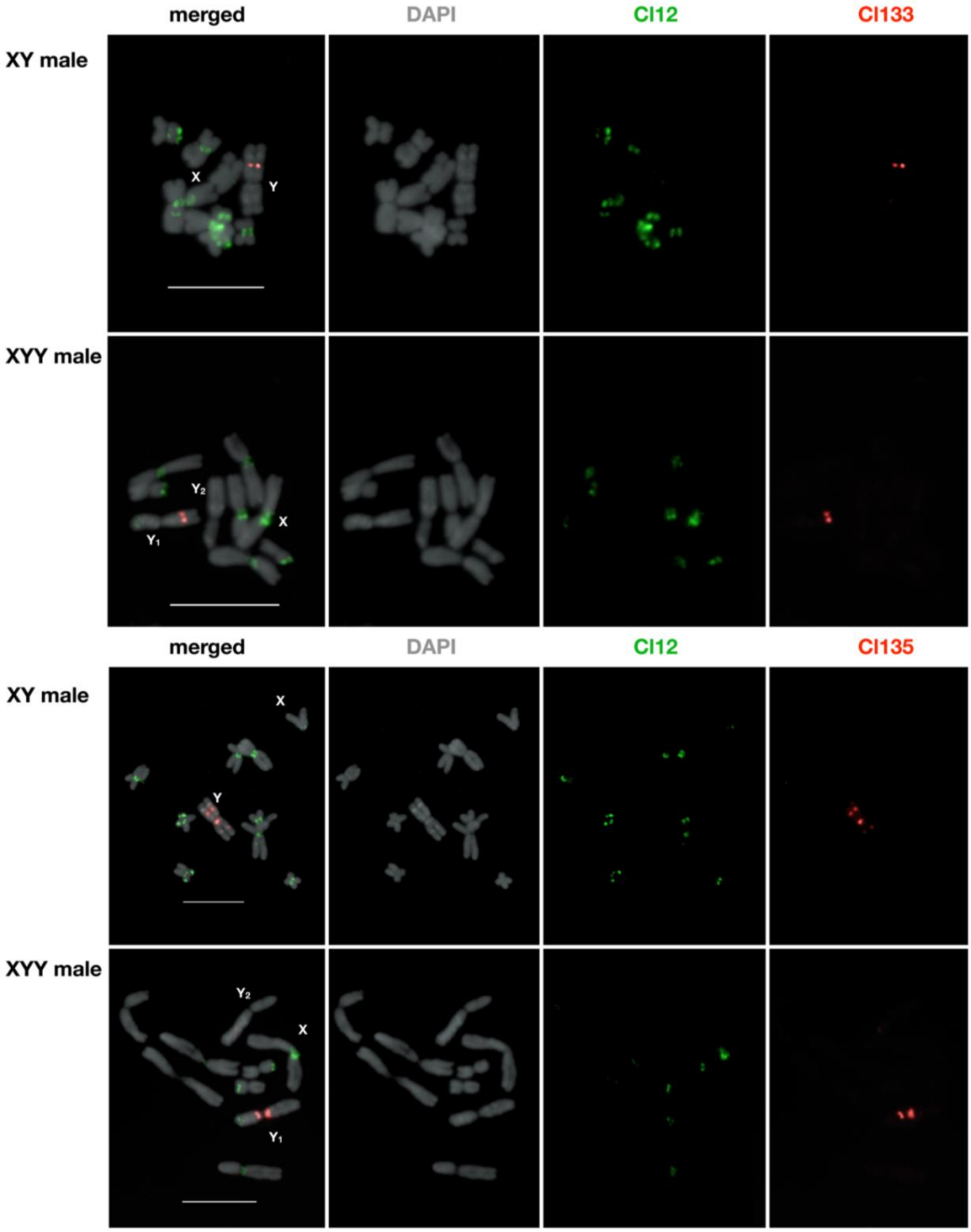

Figure S8. The localization of Cl12, Cl133 (red-top) and Cl135 (red bottom) in XY and XYY males cytotypes. Metaphase chromosomes counterstained with DAPI (grey). Scale bar = 10 mm

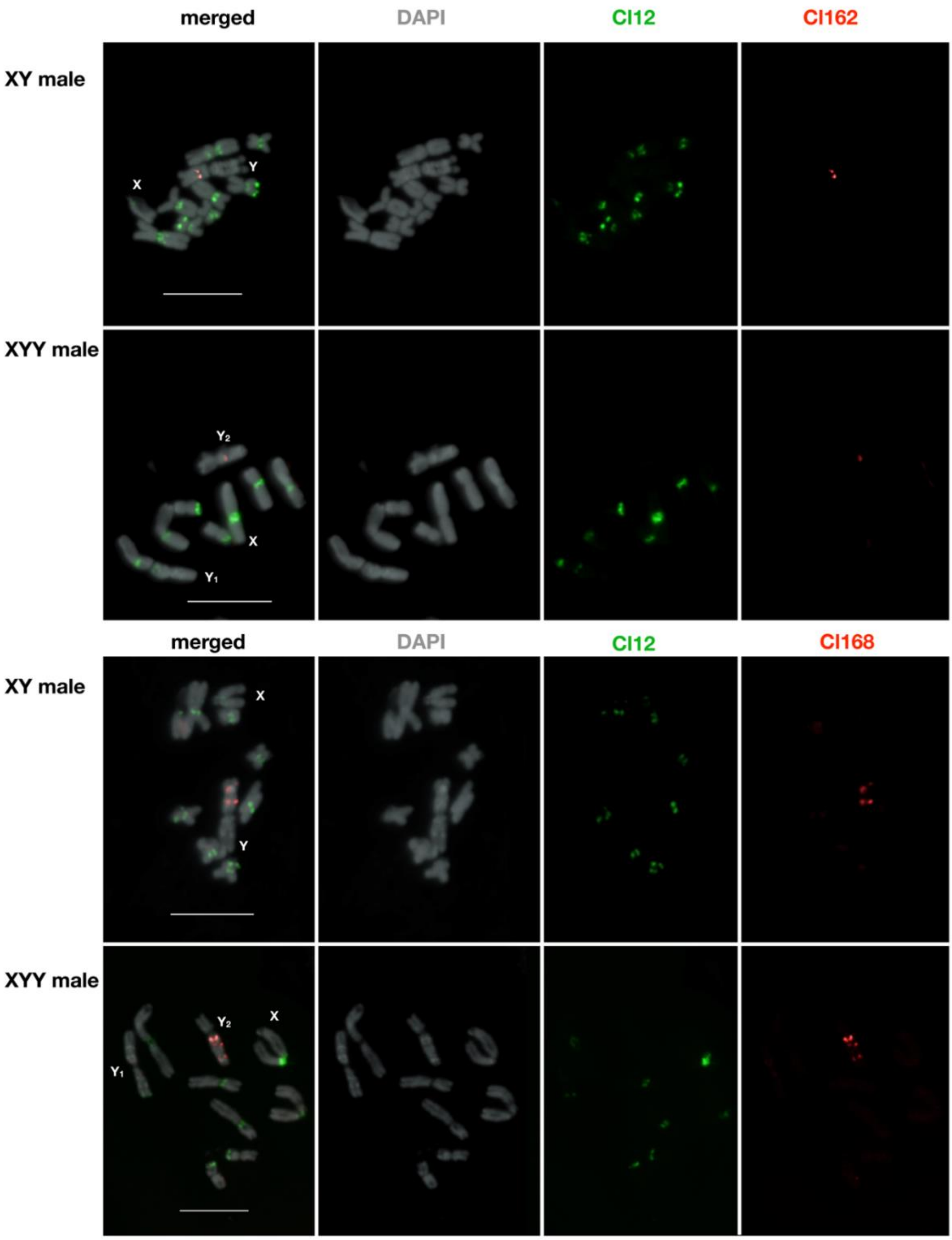

Figure S9. The localization of Cl12, Cl162 (red-top) and Cl168 (red bottom) in XY and XYY male cytotypes. Metaphase chromosomes counterstained with DAPI (grey). Scale bar = 10 mm

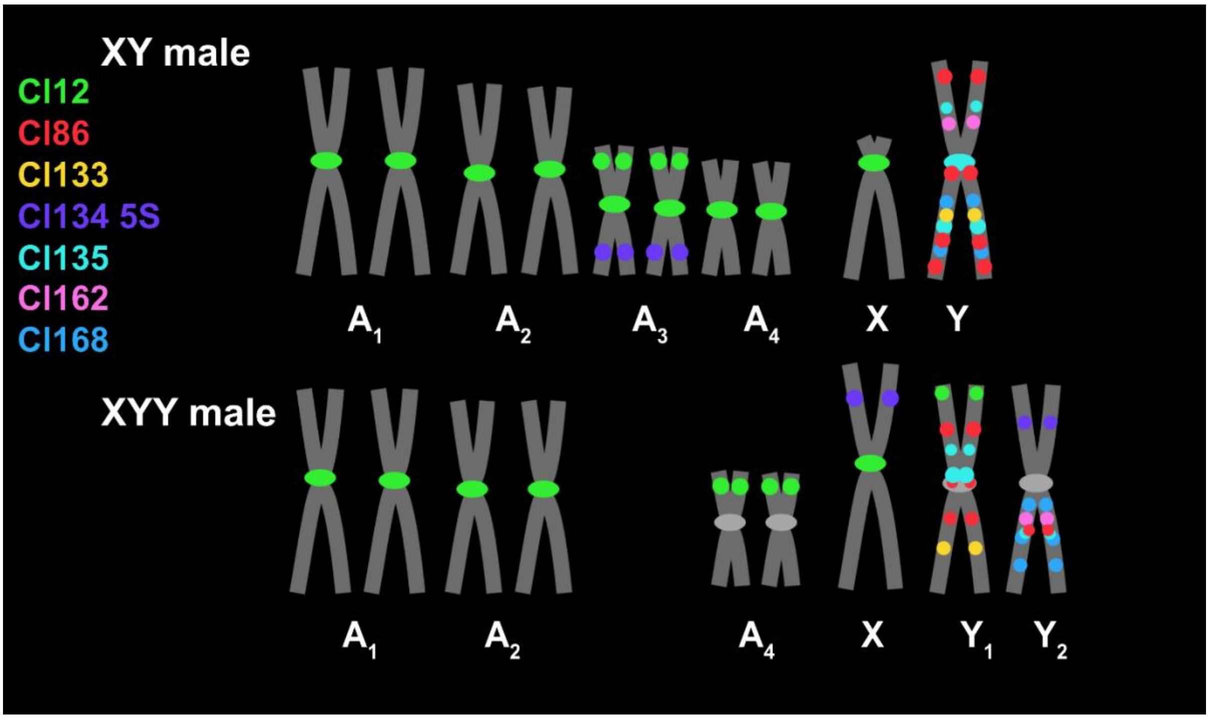

Figure S10. The scheme of satellite distribution in the XY and XYY male cytotypes of *Rumex hastatulus*. The satellite position along the chromosomal axis length is determined by ImageJ RGB plot diagram function, and its approximate order is shown above. Note different position of Cl12 between A4 in XY cytotype Texas and A4 in XYY cytotype North Carolina, confirming an autosomal inversion on this chromosomal pair.

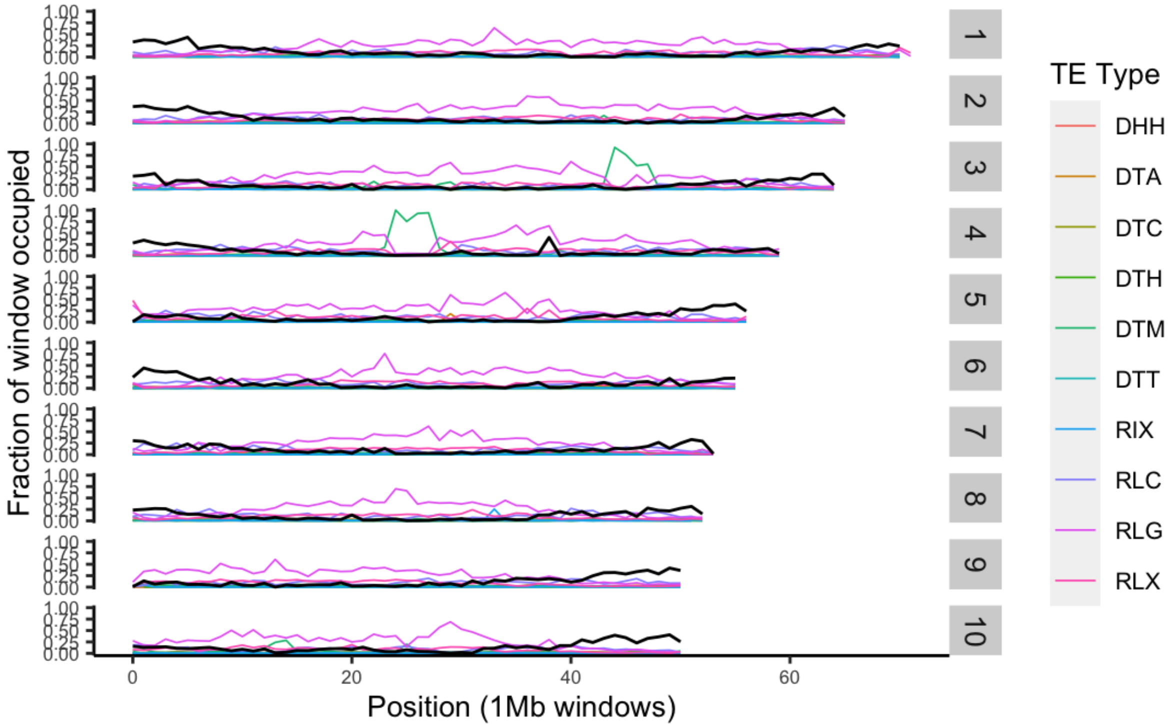

Figure S11: Proportion of 1 Mb non-overlapping windows across *Rumex salicifolius* chromosomes occupied by genes and TE sequences. Black lines are the fraction of the window that is genic sequence and coloured lines are fractions of the window that is TE sequence.

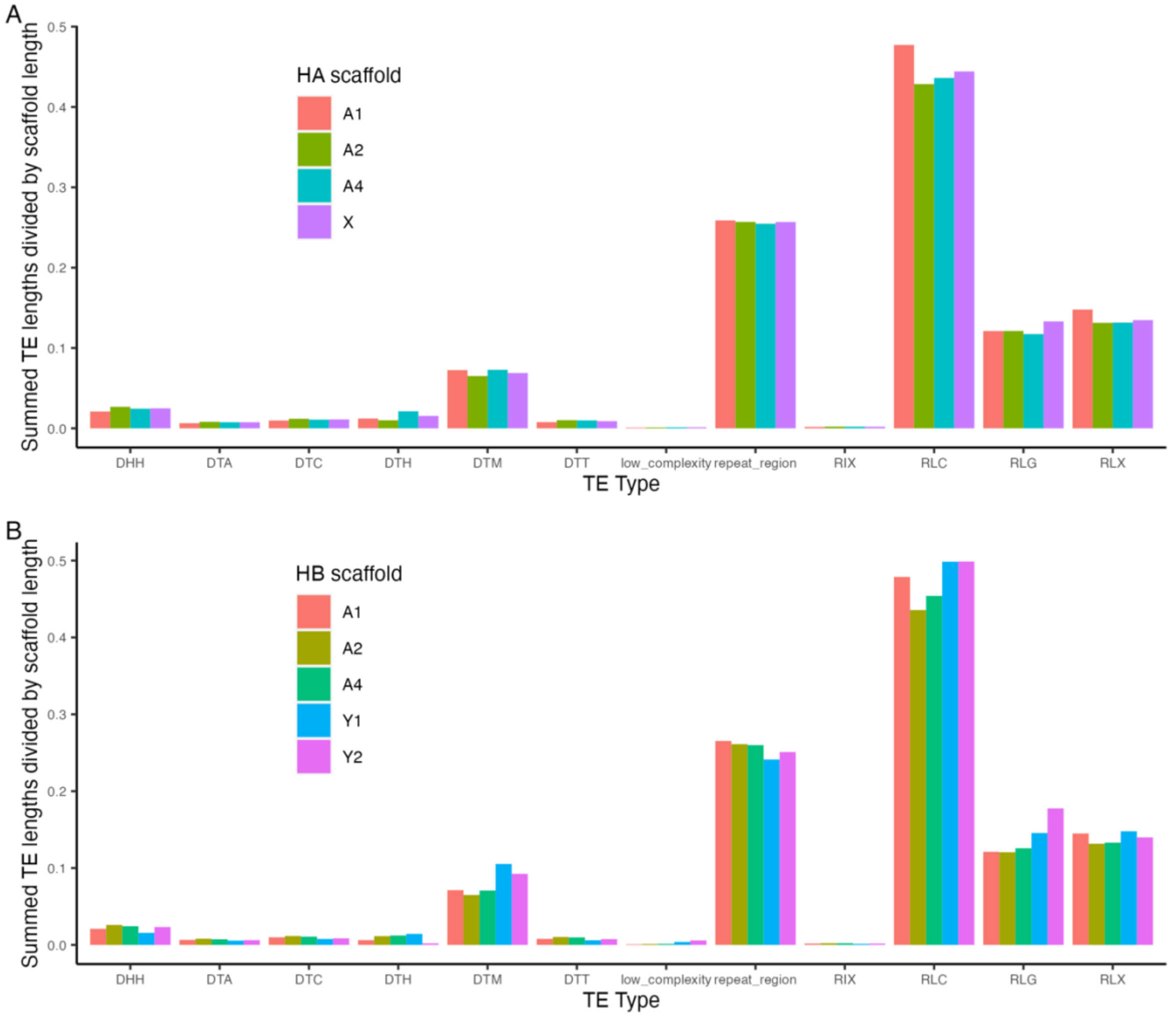

Figure S12: Proportion of *Rumex hastatulus* chromosomes occupied by TE sequence, separated by TE type. **A**: TE proportions for the X-bearing A haplotype. **B**: TE proportions for the Y-bearing B haplotype.

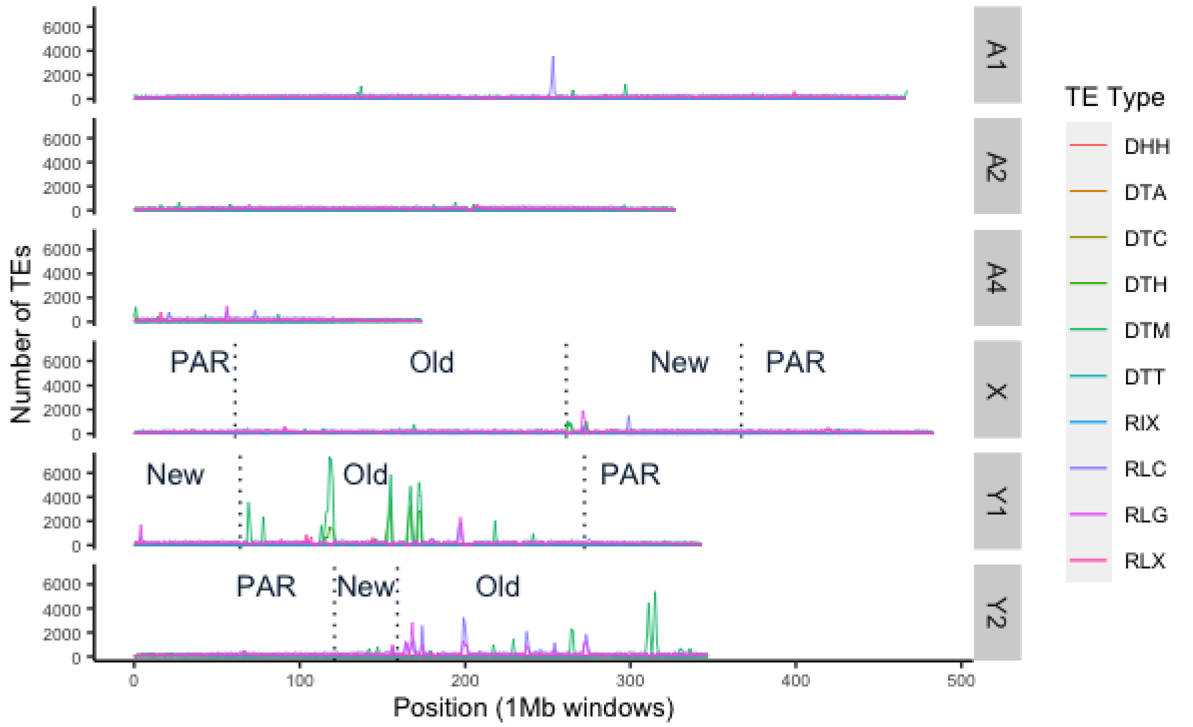

Figure S13. Chromosomal landscape of TE counts in *Rumex hastatulus*. The Y axis reflects the number of TEs for each 1Mb window, coloured by TE type.

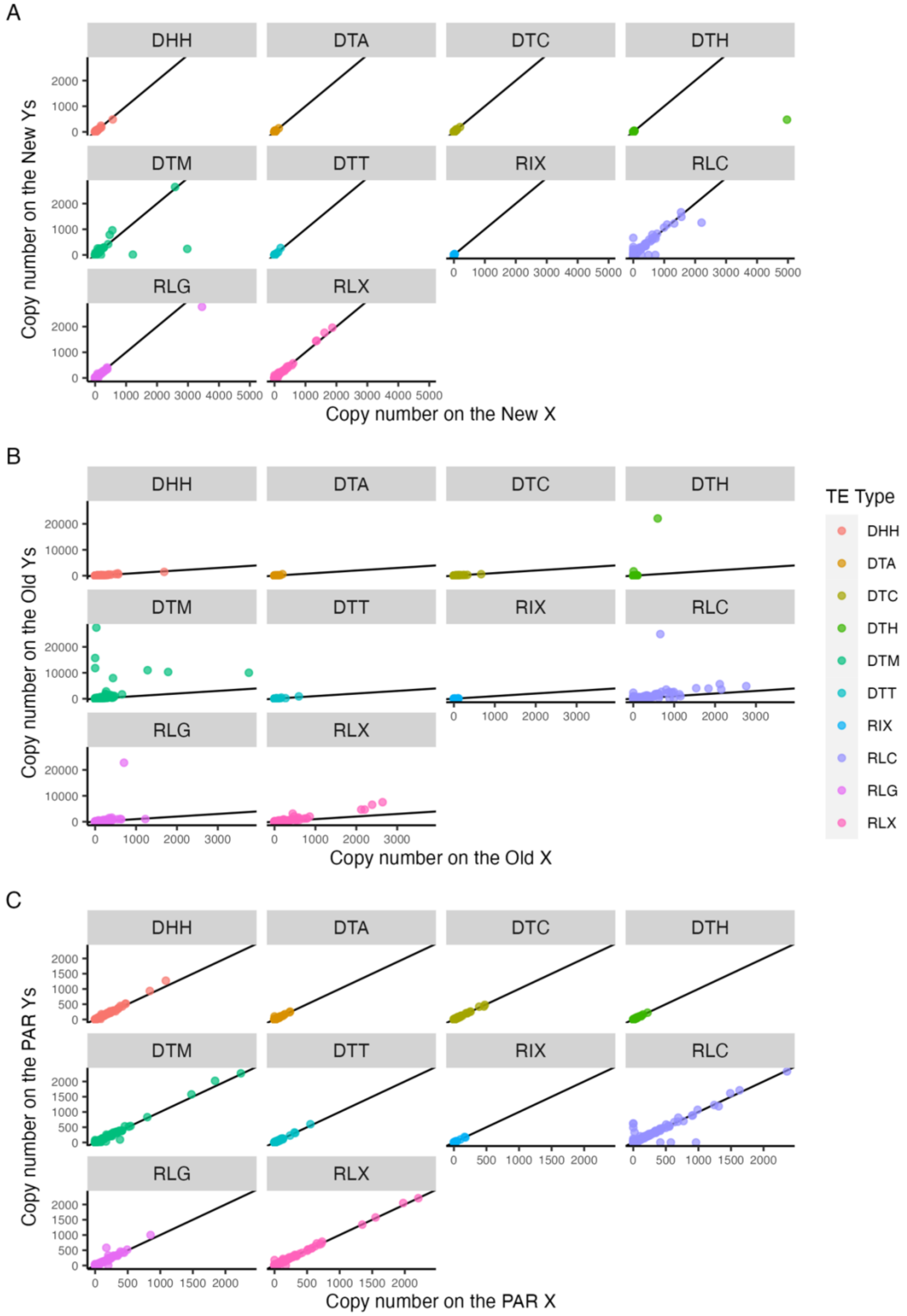

Figure S14. TE family copy number by TE Type. **A:** Comparison of TE copy number on the new sex-linked regions. **B:** Comparison of TE copy number on the old sex-linked regions. **C:** Comparison of TE copy number on the pseudo-autosomal regions (PARs). The three-letter transposon codes in the legend are from Wicker et al. 2007. DHH: helitron, DTA: hAT, DTC: CACTA, DTH: Harbinger, DTM: Mutator, DTT: Tc1-Mariner, RIX: unknown LINE (long

interspersed nuclear element), RLC: Copia, RLG: Ty3, RLX: unknown LTR (long terminal repeat)
